# Synthesizing the temporal self: robotic models of episodic and autobiographical memory

**DOI:** 10.1098/rstb.2023.0415

**Published:** 2024-09-16

**Authors:** Tony J. Prescott, Peter F. Dominey

**Affiliations:** ^1^ Department of Computer Science and Sheffield Robotics, University of Sheffield, Sheffield, UK; ^2^ INSERM UMR 1093-CAPS, Université Bourgogne Franche-Comté, UFR des Sciences du Sport, Dijon, France

**Keywords:** episodic memory, autonoetic consciousness, temporal self, cognitive architecture, predictive processing, biomimetic robotics

## Abstract

Episodic memories are experienced as belonging to a self that persists in time. We review evidence concerning the nature of human episodic memory and of the sense of self and how these emerge during development, proposing that the younger child experiences a persistent self that supports a subjective experience of remembering. We then explore recent research in cognitive architectures for robotics that has investigated the possibility of forms of synthetic episodic and autobiographical memory. We show that recent advances in generative modeling can support an understanding of the emergence of self and of episodic memory, and that cognitive architectures which include a language capacity are showing progress towards the construction of a narrative self with autobiographical memory capabilities for robots. We conclude by considering the prospects for a more complete model of mental time travel in robotics and the implications of this modeling work for understanding human episodic memory and the self in time.

This article is part of the theme issue ‘Elements of episodic memory: lessons from 40 years of research’.

## 1. Introduction

The final answer to the question of why cognitive psychology has neglected subjective experience of remembering—the answer I find most plausible—is this: the question can meaningfully arise only in the study of episodic memory. [1, p. 128-129].

  I’ve seen things you people wouldn’t believe...attack ships on fire off the shoulder of Orion... I watched C-beams glitter in the dark near the Tannhäuser Gate. All those moments will be lost in time, like tears in rain... [2, 117 minutes].

When Tulving [1,3] initiated the psychological study of episodic memory, he did so, in part, out of a desire to make sense of the relationship between memory and self, or, as he put it, to understand the ‘subjective experience of remembering’ [1, p. 128]. Forty years on from the publication of his *Elements of episodic memory*, we now understand much more about the structure, content, and development of episodic memory, but its subjective experience, or what Tulving termed ‘autonoetic consciousness’ [4], remains a relatively neglected aspect.

A similar amount of time has passed since the release of the science fiction movie *Blade Runner,* directed by Ridley Scott. Based on Philip K. Dick’s *Do androids dream of electric sheep?* [5], the film explores the nature of the human by imagining what it would mean to create a synthetic version of ourselves. The replicants in the *Blade Runner* dystopia are bioengineered, organic entities, not the electromechanical robots that exist today. Nevertheless, we consider that an embodied (robotic) computational modeling approach, or what we might call ‘synthetic psychology’ [6,7], is a promising route to understanding human memory and its relationship to sense of self [8–10]. This is the topic we will explore in this article. It remains an open question whether a synthetic entity could possess autonoetic consciousness as so eloquently captured in *Blade Runner*’s ‘Tears in Rain’ soliloquy (see [11–14] for some perspectives on this debate). Here, we will explore progress towards providing robots with capabilities similar to human episodic and autobiographical memory with the aim of casting some light on how these systems might operate in humans while also seeking to characterize what wider capacities, particularly, sense of self, would be required to create similar abilities for machines.

In §2, we first outline theories of memory and consider the distinction between episodic-like, episodic and autobiographical memory. We next provide a short summary of theories of the sense of self, focusing on the sense of self in time, for which we also discuss temporal frameworks. Finally, we briefly discuss the timeline in human development of both episodic memory and the experience of the temporal continuity of the self. Based on this review, we provide a summary position on possible relationships between self and memory in humans.

In §3, we discuss work in robotics that may cast light on the functioning and emergence of episodic memory. This section is organized as follows. We first briefly discuss robot cognitive architectures and the integration of memory systems with other components that could collectively give rise to system-level capacities such as sense of self and mental time travel. We next discuss generative models that are capable of capturing scenes as a form of multimodal content-addressable memory and that could be a useful model for thinking about memory encoding. Third, we briefly explore robot models of the self in time, highlighting the role of active inference—again, a generative approach—and consider how these might integrate with memory models. Finally, we explore models that build on linguistic capacity and that allow the construction of narrative accounts of past experience and consider how this could progress towards a more complete model of human autobiographical memory.

## 2. Memory and self

Episodic memory picks out memories of specific scenes from the rememberer’s past and can be contrasted with other forms of declarative memory that are purely semantic or factual [1,3]. For Tulving, memories are only truly episodic if they are both memories of specific scenes and are related to the self. For example, in *Elements,* Tulving quotes a passage from James’ *Psychology* (1892)— ‘Memory requires more than mere dating of a fact in the past. It must by dated in *my* past. In other words, I must think that I directly experienced its occurrence. It must have that “warmth and intimacy” […] as characterizing all experiences appropriated by the thinker as his own’ ([15, p. 650], cited in [1]). Furthermore, again following James, Tulving argued that episodic memories were more than just representations of content related to the self but carried with them a ‘characteristic phenomenal flavour of the experience of remembering’ [4, p. 2] that he termed autonoetic (self-knowing) consciousness.

It is evident from wider research in animal memory, as also discussed in other articles in this theme issue, that many animals have a scene-memory capacity that encodes the ‘what—where—when’ (www) characteristics of past experience [16]. This is often described as *episodic-like* memory. Much of this research leaves aside the question of whether animals experience such memories as being of, or belonging to, themselves. Fivush [17], in contrast to Tulving, has proposed using the term episodic memory to describe www memories, that is, memory *without* a self-component. When autonoetic consciousness is present, she advocates using the term autobiographical memory. According to her account, two further features distinguish autobiographical from episodic memory. First, that autobiographical memory provides links across multiple event memories, placing them within a temporal framework termed the ‘life narrative’, and second that autobiographical memory has a distinct social and cultural role in defining personal identity and the self in relation to others. Moreover, according to Fivush, autobiographical memory and hence autonoetic consciousness are human-specific, developing in later childhood and adolescence.

We agree that the distinction between episodic and autobiographical memory is useful (see also [18,19]), and that the latter includes significant sociocultural aspects; we will explore below how this builds on conceptual development grounded in experience and on language capacity. However, we will argue that episodic memories can be self-referential without being fully autobiographical, and that this could be based on an experience of temporal continuity and some ability to distinguish past, present, and future, rather than being further organized with respect to an ordered timeline. Moreover, as recognized by Rubin [20], aspects of autobiographical memory that are more sociocultural, such as autobiographic facts and a life narrative, also move away from the notion of memory for specific scenes, that is, they are likely to be more semanticized. We propose that distinctions between these different memory types, as summarized in table 1, are useful both conceptually and in understanding the computational processes that may be involved, as we will explore further below.

**Table 1.** Types of memory and some of their defining features. (For a discussion of whether episodic-like memory requires temporal continuity see [21].)

	scene	temporal continuity	self-referential	temporal organization	personal identity
semantic memory					
episodic-like memory	X	?			
episodic memory	X	X	X		
autobiographical memory for events	X	X	X	X	
autobiographical memory for facts		X	X	X	X

### (a) Sense of self in time

Research on the sense of self is also lacking a firm taxonomy, perhaps even more so than research on memory. A widely held view is that a *minimal self,* capable of subjective experience but lacking in reflective self-awareness, is present in many animals [22–24] and possibly in all bilaterians [25]. The key attributes of a minimal self are a sense of body ownership, including a self-other distinction, and a sense of agency, that is, an ability to distinguish outcomes that are the consequences of self-action from the broader flow of events. Most theories of the minimal self postulate that it is synchronic and not diachronic, that is, the minimal self exists in the moment and has no sense of its personal past and future [22,26]. Autonoetic consciousness would therefore not be a characteristic of the minimal self as usually conceived.

Neisser [27] postulated five different kinds of self-knowledge. The *ecological self* is the embodied self acting purposefully in the environment and perceptually differentiated from it [28]. This is broadly similar to other notions of a minimal self. Other kinds of self-knowledge, according to Neisser, are *interpersonal*, *conceptual*, *temporally extended* and *private*. Relative to the minimal self, we might consider all of these further aspects of self-knowledge as being related to the *extended self* [29]. Neisser’s temporally extended self builds on the prior development of a conceptual self, defined as a mental representation of distinguishing characteristics that persist over time and relies on an episodic memory capacity that is ‘narratively organized’ [28, p. 19].

A further substantial strand of research on self relates to the notion of the *narrative self*, defined by Dennett as the ‘center of gravity’ of the personal life story [30]. In Schechtman’s [31,32] account of the narrative self, the construction of this aspect of self builds on autobiographical narratives and on the self-concept. In other words, it bears similarities to the proposal that autobiographical memory is a sociocultural construct [17,33].

Broadly speaking then, the accounts of Fivush, Schechtman, Tulving and Neisser are aligned in viewing the existence of narrative autobiographical self-knowledge as the source of an experience of the self in time. This narrative structure also provides an organizing framework for relating scene memories to each other. However, an experience of temporal continuity could arise from other independent sources. Indeed, for Bermudez [34], forms of conceptual self, such as the narrative self, must be grounded in a pre-existing non-conceptual self in order to avoid circularity. The infant has a variety of means for judging time, such that the challenge is less about explaining the child’s timing abilities rather than how these internal timing mechanisms help give rise to cognitive capacities such as sense of self [35]. Seth & Tsakiris [36,37] have proposed that a pre-reflective experience of a diachronic self could originate in the body through monitoring of interoceptive sensory processes, including those arising from intrinsic pattern-generating systems. Specifically, the different dynamics of internally generated sensory signals, compared to externally generated ones, could lead the brain to infer the existence of the embodied self as a distinct entity (the self-other distinction) and to further infer the continuity of that self over time owing to the relative stability (and rhythmicity) of those dynamics. To distinguish this notion from Neisser’s temporally extended self, and other ideas of the continuity of self that are based on a more explicit conception of time, and/or on narrative structure, we will refer to this as the *persistent self*.

An important concept here is that of a *temporal framework* [38]. An adult experience of time is of a timeline extending from the past, through the present and into the future, onto which events can be projected as happening at particular times, with the ability to make distinctions such as between earlier and later events. This framework is conceived of as independent from events, and from the self, and is linear, ordered, directional (from past to future) and has a metric (calendar and clock time). However, a temporal framework could be as minimal as having a distinction between now and then (the past) without having any specific organization of the past. It could be grounded in the self rather than distinct from it. Moreover, we should note that a sense of temporal continuity does not require any explicit or calibrated mechanism for measuring time. As Nelson suggests, ‘all that seems to truly distinguish episodic recall from generic event memory is the sense that ‘something happened one time’ in contrast to the generic ‘things happen this way’’ [18, p. 7].

### (b) Development of episodic memory and of the sense of the self in time

Theories of episodic memory have, following Tulving, typically described this as a late-developing system (age 4+ years) that is preceded by capacities for language use and by the development of a self-concept [39]. The older child is also able to conceive of time in an adult-like way and to place memories of events in this context [38]. However, there is also significant evidence of both scene memory and temporal awareness in younger children. Specifically, children as young as 2 years can recall events in their personal past [18,40]. Two-year-olds will also use the past tense of activity verbs to refer to events that have taken place in the past, indicating that they make a distinction between current time and past time [41]. By the age of 2 years, the younger child also makes a distinction between present and past, though this is more perspectival and event-dependent than in older children [42]. Hayne *et al*. [43] have shown that 3-year-olds are able to accurately describe events in their past and to imagine plausible events in their future. These children presented their accounts in first-person terms, suggesting the presence of self-reference in thinking about past and future events.

**Figure 1.**
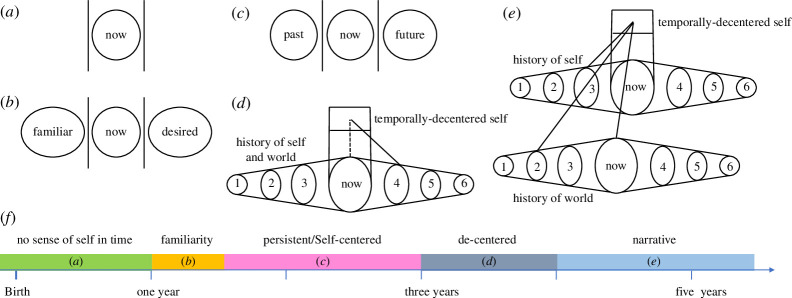
Self-consciousness in time, redrawn from [44]: (*a*) before 12 months: ‘minimal consciousness’ (see text above); (*b*) 12–18 months: ‘recursive consciousness’—familiarity with the past and desire for the future; (*c*) 18–36 months: ‘self-consciousness’—an awareness of the self as existing in the past, present and future; (*d*) 3–4 years: ‘reflective consciousness 1’—decentred and ordered sense of the self in time; (*e*) 4+ years: ‘reflective consciousness 2’—as (*d*) but the history of the self and world are distinguished; (*f*) approximate timeline of the development of sense of self in time (not part of original figure). (*a*–*e*) Copyright ©2001 from *The self in time: developmental perspectives* edited by Chris Moore and Karen Lemmon. Reproduced by permission of Taylor and Francis Group, LLC, a division of Informa PLC.

Alongside the child’s emerging awareness of culture and language, there is increasing self-awareness. However, at what point does the child conceive of herself as, in some way, the same yesterday, today and tomorrow? According to Neisser, the temporally extended self does not emerge until at least 4 years old, a proposal that aligns with Tulving’s estimate of the emergence of an episodic memory system [39]. However, as we have noted above, the construction of a simpler notion of the self as enduring in time could precede the accumulation of autobiographical knowledge and the use of narrative to organize time and memory. Zelazo & Sommerville [44] propose such an account of the development of self-consciousness in time as illustrated in figure 1. In the first year of life, they consider that the infant has a minimal form of consciousness with no temporal dimension (see also [45,46]). Around the age of 12 months, alongside other significant developmental milestones including first words, joint attention and social referencing, the infant develops ‘recursive consciousness’ in which she has an experience of familiarity for past experiences and of desire in relation to future-oriented states. From around 18 months, the child develops ‘self-consciousness’ which includes a minimal experience of the self in time that discriminates the present from past and future. From 3 years of age, the child has a form of ‘reflective consciousness’, which includes an ordering of past and future states and some capacity for temporal decentering (seeing time as distinct from the self), with adult-like decentering emerging by the age of 4–5 years.

We interpret Zelazo & Sommerville’s ‘self-consciousness’, which includes a minimal capacity to distinguish past, present and future (figure 1*c*), as indicating the presence of a persistent self in the younger child (age 18–36 months) that could support a form of autonoetic consciousness. This proposal has similarities to Howe & Courage’s suggestion of a ‘cognitive self’ [47], also from age 18 months, that includes the important capacity of the child to consider itself as an object (James’ ‘me’ rather than simply ‘I’ [15]). This proposal also aligns with the relatively slow development of parts of the hippocampal system, which matures by around 2 years old [48], and is thought to be one of the key brain substrates that supports the capacity to construct and store episodic memories [49]. Note that Zelazo & Sommerville’s timetable is also consistent with the emergence of the narrative self in the older child alongside ‘reflective consciousness’.

Table 2 summarizes the relationship between the experience of temporal continuity, sense of self and memory capacity that we have outlined so far and will explore via robotics approaches in the remainder of this article.

**Table 2.** Hypothesized relationships between possible temporal frameworks, sense of self and memory capacity. (See text for further explanation.)

development	from birth (or earlier)	from 18 to 24 months	4+ years
*temporal framework*	none (but intrinsic timing mechanisms)	self-centered	decentered
*sense of self*	minimal self	persistent self	narrative self
*memory capacity*	non-scene memory with sense of familiarity	episodic memory	autobiographical memory

## 3. Understanding memory and self through robotics

Memory is a system-level capacity of the complex system that is the human mind and brain. Hence, its understanding can benefit from the construction of embodied computational models, in the form of biomimetic robots, that integrate relevant aspects of perception, learning and representation [50,51]. The concept of episodic memory has been applied in robotics to address challenges such as planning [10,52–54], catastrophic forgetting [55] and contextualized remembering for human–robot interaction [8–10,56–60]. Here, we focus on the capacity of these models to provide insight into human memory rather than their specific use for robotics. The sense of self is also being investigated in robotics, as reviewed in [29,61]; here, we focus on models relevant to understanding the temporal continuity of the self and briefly discuss how this emerging self could impact the experience of episodic memory. In the final part of this contribution, we consider cognitive architectures that have been investigated in robotics to explore the relationship between sensorimotor activity, language, narrative and memory.

### (a) Robot cognitive architectures

An important characteristic of many robot control systems is the use of layered cognitive architectures [62–65]. Layering provides the capacity to co-ordinate responses on different timescales, with different forms and depths of internal processing, and provides robustness through the presence of multiple solutions [51,66]. Layered architectures are also characteristic of vertebrate brains and have evolutionary and developmental trajectories [63,65]. The emergence of consciousness can be understood in relation to the concept of layered control [64,67] and the association of minimal consciousness with upper brainstem structures that are mature at birth [45]. The emergence of self- and reflective consciousness in the early years (as discussed above) therefore reflects, in part, the maturation of cortical systems, while lower-level circuits continue to function. The emergence of temporal cognition has been explored from a similar dual-system perspective [21].

### (b) Episodic memory systems in robotics

**Figure 2.**
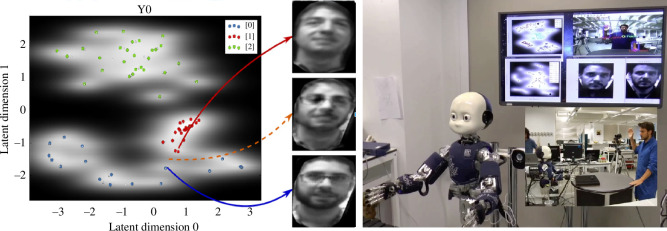
Generative modeling of memory using Gaussian process latent variable models (GPLVMs) illustrated with an example of face learning. Left: high dimensional images are encoded in a two-dimensional latent variable space. Latent variables are inferred underlying constructs that describe data in a compact way and are often discovered using statistical procedures such as factor analysis. In this model, latent variables are discovered through a machine learning method that also adds the potential for nonlinear dimensionality reduction. In the figure, clusters of anchor points that encode memories of three specific individuals are colour-coded for display purposes. Lighter areas indicate higher certainty for recognising different individuals. The face images shown are reconstructions from three locations in the space as indicated by the colored arrows. The orange dotted line shows the reconstruction of a face that merges features from two actual people, demonstrating a capacity for ‘imagination’. ©2016 IEEE from [74], reprinted with permission. Right: the iCub robot using face and action GPLVMs during a human–robot interaction experiment. The combined model can recall which action was performed by whom. The relation to the self was not specifically encoded. Reprinted from [8] under Creative Commons CC-BY license.

There have been a wide range of approaches to modeling episodic memory, with greater or lesser emphasis on modeling neurological substrates and psychological findings (for reviews, see [68,69]). Recent research has demonstrated the use of generative models in capturing the capacity of episodic memory to reconstruct rich multimodal scene memories of past experience and to imagine future experience [70–72]. Key properties of such models include compression, pattern completion and pattern separation, which serve to meet the requirements for storing scene memories efficiently and retrieving them with a partial cue [73]. Prescott and colleagues [8,74,75] investigated deep Gaussian process models that learn low-dimensional representations of patterned data by constructing latent variable spaces in multiple sensory modalities. Such models can also span between modalities and efficiently represent a temporal dimension, making them suitable for compactly representing events, such as actions on objects, that unfold over time [76]. As implemented for the iCub humanoid robot and illustrated in figure 2, this model operated in both an involuntary recall mode, where remembering was triggered by sensory events, and in a voluntary retrieval mode in which patterns were retrieved using specific cues [8]. As previously noted, in the human brain, the construction of scene memories is specifically associated with the extended hippocampal system [19,49], however, imaging data suggest that modality-specific memory elements are encoded elsewhere in cortex including in primary sensory areas [77]. This notion that an episodic memory is filled out using semantic memory systems has featured in several recent models (e.g. [72]). The reconstruction of past events may also make use of the brain’s capacity for internal simulation [78]. This was modeled by Prescott *et al*. [8] by using the iCub robot’s three-dimensional physics simulator, populated with additional graphic elements drawn from recall (see also [79]).

### (c) Robot modeling of the emergence of self

**Figure 3.**
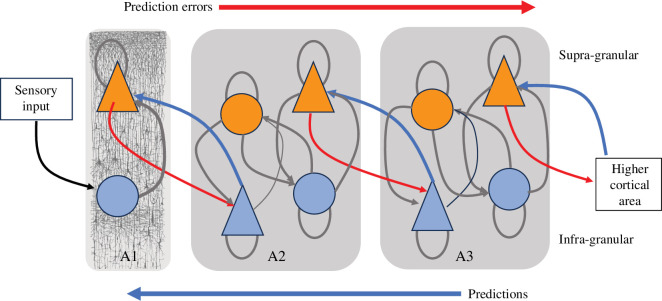
Human cortex considered as a hierarchy of generative models for predictive inference, based on [85]. Within each cortical area, or macro-column, lower layers (infra-granular) are state units and higher layers (supra-granular) are error units. Triangles denote sources of feedback and feedforward connections. Predictions adapt to minimize/cancel ascending prediction errors. See [85,86] for further explanation and [82] for a theory of self as a hierarchical model constructed through prediction error minimization and the propagation of inferences. Adapted from [85] with permission from Elsevier.

A growing literature proposes that the self is an internal model constructed by the mind in order to explain patterns of regularities that are observed while interacting with the world [80–84]. This idea is increasingly expressed in terms of hierarchical generative models that employ free energy minimization to align model predictions with incoming sensory signals [85], known as *perceptual inference*, as illustrated in figure 3. According to this *predictive processing* approach, behavior can also be seen as a form of *active inference*, whereby the agent acts so as to shape its sensory inputs and thereby minimize prediction error. Following this framework, different aspects of self can be seen as deriving from the capacity to predict various kinds of sensory signals (see Hohwy & Michael [82] for a fuller discussion). For example, agency can be viewed as the capacity to predict the content of sensory signals based on self-movement (particularly, efference copy signals) [81], while the experience of body ownership could arise from the prediction of interoceptive and proprioceptive signals [37] and self-recognition (e.g. in a mirror) from the capacity to predict exteroceptive signals that are self-generated [84].

These proposals provide a practical challenge for robotics to demonstrate that appropriate models can be inferred from sensorimotor experience [87]. Recent work by Lanillos & Cheng has applied generative modeling and free energy minimization to enable a robot to infer its own body configuration from sensory data (proprioception, visual and tactile) [88] and to recognize its own body parts in the visual scene [89]. That these capacities can support a form of self-other distinction has been demonstrated through the successful performance of a version of the mirror self-recognition test [90].

Within the predictive processing framework, which can also be related to theories of layered control [65], there is a progression from unimodal to multimodal and amodal representations, as the hierarchy of models is ascended [36,83]. There is also a corresponding broadening of temporal scales. Thus, although the persistence of the self has not been specifically modeled in robotic systems, the inference to a self that provides the high-level cause for sensory predictions would be one that transcends sensory processes and endures over time (for instance, in the form of prior beliefs). Self-consciousness, on the other hand, is a more momentary affair, potentially deriving from the focus of the current mental state towards the task at hand or towards thinking about the future [81]. In this context, Hohwy [81] has proposed that a ‘proto-narrative self’, an early form of reflective self but without specific narrative structure, emerges from the capacity to disengage from the current activity and plan future behavior.

### (d) Robot modeling of autobiographical memory and the narrative self

Mental time travel, both into the past or the future, places interesting requirements on the underlying system, in particular, there is some notion of ‘making sense’ and structural coherence that applies to the interpretation of experience [91]. This notion of making sense has been addressed in multiple forms in the context of narrative. For instance, Ricoeur [92] has argued that adult perception is prefigured by a social conceptual system for understanding goal-directed actions that allows us to distinguish the domain of action from that of physical movement—‘If, in fact, human action can be narrated, it is because it is always already articulated by signs, rules, and norms. It is always already symbolically mediated.’ [92, p. 57]. Thus, as the child becomes increasingly embedded in, and aware of, her culture, perception is increasingly filtered by a system that is sensitive to the acquired norms of goal-directed action. Mandler [93] evokes a similar perspective in which image schemas create an interface between raw continuous perception and discrete language. Feldman [94] addresses this with the notion of construal. She notes that the child is not a passive copier of reality but rather constructs her own representation of the world, and that language becomes the general-purpose symbolic tool that allows the creation of versions of the world. In this final section, we explore how these principles can assist perceptual and memory systems for robots in making the transition from the uninterpreted flow of sound files and megapixel camera frames into a meaningful conceptual world of motivated, goal-directed events.

Goal-directed action can be characterized in terms of an initial state, some sequence of perceptual changes and a final state. Following earlier work showing that dynamic primitives such as contact, support or attachment can be extracted from video data and used to recognize actions on objects [95], Dominey & Boucher [96,97] developed a robotic model that exploited the observation that infants are sensitive to physical contact between objects [98]. They hypothesized that actions involving object transfer (such as push, give and take) could be characterized as sequences of perceptual contact events and built an event parser that transformed sequences of contact events into predicate-argument action representations [96,97]. Learning the mapping between sentences and the action representations of the situations they describe resulted in the development of a structured set of sentence form-to-meaning mappings, in other words, simplified grammatical constructions. Importantly, this structural link between action representations and corresponding grammatical constructions allowed the system to learn novel action representations and to communicate about them [99].

Building on this approach, Pointeau *et al*. [100] developed an autobiographical memory system for the iCub humanoid that recorded experienced events as episodic memories that could be indexed by time and included relevant context (who, what, where, when). These experiences could then be enriched and given structure by language to allow the emergence of semantics about locations, spatial and temporal relationships, actions and shared plans. By storing experience, the memory model provided the basis for time travel into the past. Knowledge about actions included initial states and resulting states, and this information could be used to travel forward in time. For example, a specific form of mental time travel into the future was realized when Pointeau *et al*. took advantage of a forward model that was used for short-term predictions in the context of motor control. By extracting this model from the specific case of online control and allowing it to have the status of a mental representation, the model could be used as a mental simulator to observe the potential consequences of future actions [79].

While robotic systems can extract different kinds of structural regularities from a camera image (e.g. related to object positions and object-human contacts), it is the labels provided by language and by a human teacher that bring them into the shared space of interaction. As noted by Feldman [94], the same scene can be construed in different ways, and one function of language is to establish the desired construal. Building on the grammatical constructions of visual scenes developed in the earlier study, Mealier *et al*. [59] demonstrated that the same scene could be associated with multiple event representations emphasizing, for example, the action performed or the result of that action (‘John gave the book to Mary’ vs ‘Mary has the book’), showing that language could be used to create these different construals.

**Figure 4.**
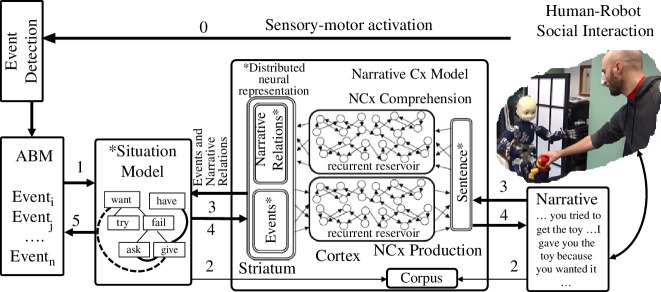
Cognitive system for the iCub robot that allows representations of events to be enriched by language using a situation model and a synthetic autobiographical memory (ABM). Propositional representations are extracted from the visual scene by event detection and perceptual processing (0), encoded in the ABM, and then converted into state—action—state representations in the situation model (1). The human narrates the interaction (2) and sentences in that narrative are aligned with the situation model to generate a corpus of sentence-meaning pairs that is used to train comprehension and production models (3) implemented using reservoir computing (a form of recurrent neural network model inspired by cortical language circuits [102]). The trained model allows relational links coded in the narrative to enrich the situation model (3). Once a situation model has been instantiated, the system extracts the meaning to the narrative production model to generate the corresponding narrative (4) and to enrich the ABM (5). New experiences that resemble learned situation models can therefore inherit enriched narrative structure. Taken from [60] which should be consulted for more details. A version of this model was integrated within a layered robot cognitive architecture in [10]. Reprinted from [60] under Creative Commons CC-BY license (CC-BY 4.0).

Fivush *et al*. [101] have argued that in order for experience to be encoded in autobiographical memory, that experience must be encoded in a narrative. This poses non-trivial challenges for building robotic systems. Mealier *et al*. [60] developed a cognitive framework to begin to explore the constructions of robot memories that are enriched and made coherent through a mapping with narrative structure. As illustrated in figure 4, this system could learn mappings between narrative patterns and a structured collection of perceived events in episodic memory and demonstrated a simple form of time travel into the past. The model displayed a powerful one-shot learning for the mapping between situation models and narrative patterns. The weakness of this method was the relatively brittle structure of the narrative patterns that were learned in a single shot. To overcome this, Pointeau *et al*. [79,100,103] developed a machine learning approach that learned regularities about how people narrate perceived actions. This predictive knowledge was then employed by the robot, enabling a more flexible capability to narrate past actions that indicated a form of mental time travel to the past.

More recently, a robotic episodic memory capability that combines latent variable encoding with natural language question-answering capabilities has been developed by Bärmann and colleagues [57] for the ARMAR III humanoid. This model combined an episodic memory model based on a convolutional neural network auto-encoder, which generated low-dimensional representations of robot interaction episodes [54], with a speech encoder–decoder based on a transformer network model [104]. The model also included a component that could estimate a probability distribution over encoded time information. The robot was able to answer natural language questions about past events, although performance degraded with the number of events stored.

The above summary demonstrates progress towards creating synthetic episodic and autobiographical memory for robots, highlighting efforts that address related challenges including building a sense of self for robots and creating a grounded language capacity that can be used to structure, abbreviate and interrogate encoded memories. We note that progress in generative modeling has led to some significant breakthroughs and suggests candidate solutions for multiple parts of the puzzle. However, existing systems are clearly partial and individually address only some aspects of the challenge—in robotics, the problem of integrating multiple capabilities in a functional cognitive architecture should not be underestimated.

## 4. Discussion and concluding remarks

We have presented some proposals for the incremental emergence of episodic memory alongside a sense of self and have provided evidence of how aspects of these proposals could be fleshed out through a synthetic psychology approach.

One core idea is the construction of layered architectures, such that multiple solutions to a problem (memory, time or sense of self) can co-exist and have different evolutionary and developmental trajectories. A second key principle is that brains seek to predict, explain and structure their own sensory inputs by constructing generative models. We have seen how this idea can be applied to develop powerful forms of content-addressable memory and to allow a robot to infer its own body and agency. In recent years, powerful generative artificial intelligence (AI) methods, including convolutional neural networks, transformers and diffusion models, have led to a step-change in our ability to model perceptual and language processing [105] and are likely to further extend our capacity to create memory systems and self-models for robots.

Dual process theories (e.g. [106,107]) suggest distinct brain circuits for regenerating an encoded memory (recall) and determining whether it should be identified with the past (familiarity). However, the predictive processing approach suggests a wider view that ‘pastness’ or ‘mineness’ happens as causal inferences propagate through the brain to reduce prediction errors. Thus, priors that connect sensory signals to the body or to past experience might arise from many different brain processes, indeed, from anywhere in the body (on the basis that the whole agent, and not just its brain, is the model [108]). Remembering, which is itself a form of prediction, will therefore be, in part, a bodily state, and this wider pattern is what we should understand as giving rise to the experience of familiarity or self-relatedness. In other words, if we are to look for the neural correlates of autonoetic consciousness—James’ ‘warmth and intimacy’—this will probably involve the more body-oriented and visceral aspects of our nervous systems [24,36], and the brainstem substrates of consciousness [24,67] that are present in our earliest years [45]. Moreover, in our view, if we are to create artefacts that have their own phenomenal experience of remembering, they will also need bodies of some kind and layered cognitive architectures, that is, they will need to be a particular kind of embodied (robotic) AI (see also [12,64]).

At the more abstract levels of the mind’s generative hierarchies, we can expect to find concepts, language, cultural constructions and anticipated regularities in social behavior that can be characterized in terms of folk psychology [91,109,110]. From a predictive processing point-of-view, the construction of a life narrative can be seen as a further way to organize experience into patterns that are meaningful, coherent and reduce uncertainty [111]. In his concept of the narrative construction of reality, Bruner [91,110] has argued that we organize our experience into a coherent shared narrative, which not only describes reality but actually constitutes it. The shared reality that we co-construct is not necessarily the most veridical one [37] rather the one that most successfully predicts our interactions as co-operative social beings.

Theories of self, like theories of memory, are difficult to make tangible. In advocating a synthetic approach, our goal is to make theoretical notions concretely embodied and observable [112]. Tulving himself was skeptical that an AI could possess episodic memory (not so semantic memory) [1]. Indeed, the deficits noted by Tulving—lack of temporal organization, sense of personal identity and continuity in time—accurately describe some of the limitations of most current AI and robotic systems. However, as we have explored in this article, there are paths forward that could yield synthetic entities with a capacity for episodic memory and mental time travel. Further progress may help us better understand our own relationship with the lived past and the subjective experience of remembering.

## Data Availability

This article has no additional data.
